# PR Toxin – Biosynthesis, Genetic Regulation, Toxicological Potential, Prevention and Control Measures: Overview and Challenges

**DOI:** 10.3389/fphar.2018.00288

**Published:** 2018-03-29

**Authors:** Manish K. Dubey, Mohd Aamir, Manish S. Kaushik, Saumya Khare, Mukesh Meena, Surendra Singh, Ram S. Upadhyay

**Affiliations:** ^1^Centre of Advanced Study in Botany, Institute of Science, Banaras Hindu University, Varanasi, India; ^2^Department of Biochemistry, Institute of Science, Banaras Hindu University, Varanasi, India; ^3^Centre for Transgenic Plant Development, Department of Biotechnology, Faculty of Science, Hamdard University, New Delhi, India

**Keywords:** *Penicillium roqueforti*, mycotoxicosis, detection, prevention, biosafety, impact and legislation

## Abstract

Out of the various mycotoxigenic food and feed contaminant, the fungal species belonging to *Penicillium* genera, particularly *Penicillium roqueforti* is of great economic importance, and well known for its crucial role in the manufacturing of Roquefort and Gorgonzola cheese. The mycotoxicosis effect of this mold is due to secretion of several metabolites, of which PR toxin is of considerable importance, with regard to food quality and safety challenges issues. The food products and silages enriched with PR toxin could lead into damage to vital internal organs, gastrointestinal perturbations, carcinogenicity, immunotoxicity, necrosis, and enzyme inhibition. Moreover, it also has the significant mutagenic potential to disrupt/alter the crucial processes like DNA replication, transcription, and translation at the molecular level. The high genetic diversities in between the various strains of *P. roqueforti* persuaded their nominations with Protected Geographical Indication (PGI), accordingly to the cheese type, they have been employed. Recently, the biosynthetic mechanism and toxicogenetic studies unraveled the role of *ari1* and *prx* gene clusters that cross-talk with the synthesis of other metabolites or involve other cross-regulatory pathways to negatively regulate/inhibit the other biosynthetic route targeted for production of a strain-specific metabolites. Interestingly, the chemical conversion that imparts toxic properties to PR toxin is the substitution/oxidation of functional hydroxyl group (-OH) to aldehyde group (-CHO). The rapid conversion of PR toxin to the other derivatives such as PR imine, PR amide, and PR acid, based on conditions available reflects their unstability and degradative aspects. Since the PR toxin-induced toxicity could not be eliminated safely, the assessment of dose-response and other pharmacological aspects for its safe consumption is indispensable. The present review describes the natural occurrences, diversity, biosynthesis, genetics, toxicological aspects, control and prevention strategies, and other management aspects of PR toxin with paying special attention on economic impacts with intended legislations for avoiding PR toxin contamination with respect to food security and other biosafety purposes.

## Introduction

Microbial contamination, particularly with filamentous fungi, could result in the production of mycotoxins whose consumption may have drastic implications on the health of both animals and humans, and is the one of the most important and major concern, for all that involved in food safety issues (Adeyeye, 2016). The molds responsible for contamination of food and other dairy products are highly diverse (both at the genus and species level) and frequently employed in the various industrial application for processing of feed and food products. The dairy products that flourished with the *Penicillium* spp. includes blue-veined, mold-ripened, hard and semi-hard cheeses, softer- and semi-soft cheeses, butter or yogurt as well as other milk-based derivative products (Garnier et al., 2017). Out of the various mycotoxigenic species of *Penicillium*, the mold that belongs to *Penicillium roqueforti* complex having four species that includes *Penicillium roqueforti, P. carneum, P. paneum* and *P. psychrosexualis* (Frisvad et al., 2004; Houbraken et al., 2010; Houbraken and Samson, 2011), have been reported to be most dominant post-harvest fungi, and preferably grow in forages/silages under the microaerophilic, moderately acidic and psychrophilic conditions (Pereyra et al., 2008; Storm et al., 2010; Driehuis, 2013; Wambacq et al., 2018). The secondary metabolites produced by various *penicilli* include severe mycotoxins such as penicillic acid, isofumigaclavines A and B, festuclavine, roquefortine C, PR toxin (Fontaine et al., 2015; Gillot et al., 2017a), and eremofortins (EreA-E) (Chang et al., 1998) and other bioactive compounds such as andrastin A, and mycophenolic acid (García-Estrada and Martín, 2016). The secondary metabolites (mycotoxins) associated with these fungi cause acute and chronic toxicity and were reported to possess mutagenic/genotoxic, teratogenic, carcinogenic, and immunotoxic properties. Food quality and safety have become the major issues among the world growing population due to the increased public interests in health issues and rigorous demand for hygienic and quality enriched food products. Today, the consumption of contaminated food and feedstuffs resulted in various disease outbreaks and considered as a recurring problem worldwide. Although, the growth of molds on cheese surface represents the sign of microbial contamination (Sengun et al., 2008). Nevertheless, some molds having low toxigenic potential are widely used for preparing soft molded speciality cheeses with having different organoleptic characteristics such as mold-ripened cheeses. The mold-ripened blue cheeses such as Roquefort, Gorgonzola, Gammelost, and Danish Blue are manufactured by *P. roqueforti* and *P. camemberti* imparts characteristic texture, blue-green spots, and specific aroma to these cheeses. Additionally, these fungi add a unique flavor to the food products, protect them against unwanted contaminants, and give the desired color.

*Penicillium roqueforti* is one of the most common blue-green sporulating fungi, frequently found in silages (Hymery et al., 2017) and various other matrices like refrigerated stored foods e.g., shredded cheese, meats, bakery products, other wheat, rice and maize products (Lund et al., 1996; Boysen et al., 2000; Lavermicocca et al., 2003; Pitt and Hocking, 2009; Storm et al., 2010; Ropars et al., 2012; Martín and Coton, 2017). The fungus *P. roqueforti* possesses many favorable and ambivalent characteristics such as favorable growth at moderately acidic pH, low O_2_ and high CO_2_ level (microaerophilic), and under psychrophilic conditions (Hymery et al., 2017). Additionally, many *P. roqueforti* strains have been reported that were found to be tolerant to weak preservatives, an even well flourished in 0.5% acetic acid and 9000 ppm. The fungal growth is well favored and stimulated at low salt concentrations, with 1% salt (NaCl) inducing the maximum stimulating effect. The biochemical machinery (enzymatic system) in this fungus has been well characterized, and revealed the presence of efficient proteolytic and lipolytic enzymes (for cheese ripening and flavor production), by utilizing both hexoses and pentoses as substrate. These favorable characteristics allow this fungus to be employed as starter culture, maturating agent in all varieties of blue cheeses (Fernández-Bodega et al., 2009; García-Estrada and Martín, 2016) and considered as an ideal choice for various commercial exploitation in the industrial biotechnology sector.

During the cheese manufacturing process, the profound NaCl gradient developed from the core to the surface of blue cheeses provides favorable microenvironment, which slowly reaches to the equilibrium during cheese ripening, and therefore, supports the germination, sporulation, and growth of *P. roqueforti* over other *Penicillium* spp. for the manufacturing of cheese. It has been reported that many *P. roqueforti* strains, isolated and cultured from different sources like commercial blue cheeses, moldy grains and nuts have eminent potential to produce mycotoxins such as PR toxin, penicillic acid, isofumigaclavin C, patulin, roquefortine and botryodiploidin in laboratory cultured conditions (Jong and Gantt, 1987). However, the three most common mycotoxins secreted by *P. roqueforti* include mycophenolic acid (MPA), PR toxin, and roquefortine C (ROQ C) (Rasmussen et al., 2010; Gillot et al., 2017a; Martín and Coton, 2017).

**FIGURE 1:**
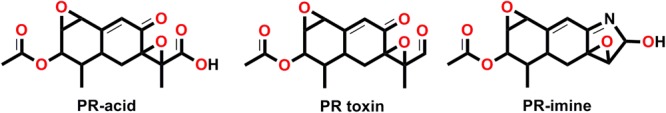
Structure of PR toxin and its derivatives.

The occurrences and contamination of PR toxin (7-acetoxy-5,6-epoxy-3,5,6,7,8,8a-hexahydro-carboxaldehyde), a bicyclic sesquiterpene that belongs to eremophilane on cereal, maize, forages/grass silages (O’Brien et al., 2006; Rasmussen et al., 2010), and cheeses (Scott et al., 1977; Scott, 1981; Fernández-Bodega et al., 2009) have been well documented. However, the report on blue cheese contamination with PR toxin is less available due to their degradation or unstability. Based on the studies done on laboratory animals, it was shown that PR toxin is of greater toxicological concern, but found to be unstable and get converted into its substituted and less toxic derivatives (Hymery et al., 2014) such as PR imine (Siemens and Zawistowski, 1993), or could be degraded into PR amide and/or PR acid (depending on the conditions available) during the cheese manufacturing at low O_2_ concentrations (**Figure 1**; Chang et al., 1993, 1996, 2004a,b). In contrast, roquefortine, isofumigaclavine A, mycophenolic acid and the siderophore ferrichrome have been detected in blue cheese at low ppm levels. Furthermore, like other metabolites, the quantitative amount of PR toxin production in all the reported strains of *P. roqueforti* is not common for all the isolates and has been shown to have highly strain dependent production variabilities.

The PR toxin producing *P. roqueforti* was first isolated and partially characterized by Wei et al. (1975). The biosynthetic cluster involved in PR toxin production in *P. roqueforti* was recently elucidated (Hidalgo et al., 2014, 2017). The biosynthetic route, leading to the production of PR toxin have been confirmed through various precursors (^14^C and ^13^C) and revealed the isoprenoid route for biosynthesis of PR toxin (Pedrosa and Griessler, 2010). Eremofortins (Ere), and PR toxin are closely related with the hydroxyl group (-OH) in Ere being replaced by aldehyde (-CHO) group in PR toxin at C-12 positions and crucial for bioconversion of Ere to PR toxin (Pedrosa and Griessler, 2010). The chemical changes that leads into the formation of PR toxin is the condensation and cyclization of farnesyl-diphosphate (three molecules), catalyzed by aristolochene synthase (encoded by the *ari1* gene) (Hohn and Plattner, 1989; Cane et al., 1993; Proctor and Hohn, 1993). It was experimentally demonstrated that PR toxin is the most toxic metabolite secreted by *P. roqueforti* strains causing significant damages to the liver, kidney and has potential for mutagenicity, carcinogenicity, *in vivo* inhibition of DNA replication, transcription and protein synthesis (Moulé et al., 1976, 1978; Ueno et al., 1978; Polonelli et al., 1982). The PR toxin secreted by *P. roqueforti* was reported to be most acute and toxic metabolite among the other metabolites secreted by the fungus with respect to the human health (Polonelli et al., 1978; Storm et al., 2014; Hymery et al., 2017) as it was demonstrated that exposure of PR toxin on the intestinal Caco-2 and/or THP-1 cells induces necrosis and an inflammatory response (Hymery et al., 2017) and possesses broad spectrum biochemical activities causing toxicosis in animals. The major outbreaks due to PR toxin-induced mycotoxicosis effect in animal feeds were reported (Le Bars and Le Bars, 1989; Scudamore and Livesey, 1998). Since, the level of this mycotoxin in food and feed products are not regulated, the contamination of *P. roqueforti* in grass silages, grains, or other food and feedstuffs could have a safety risk, and could not be deteriorated using normal cooking processes, therefore, potentially have both health and economic impacts (Kumar et al., 2017).

The assessment of mycotoxins present in food and feed products is necessary for determining the nutritional values of the consumables and other derived products. Additionally, the cytotoxic response of the consumed mycotoxins is of utmost importance to determine the detrimental limits and to explore the novel approaches for mitigating the toxic effects of toxin-producing strains. Several physical, chemical, and biological approaches have been employed for mycotoxin assessment in recent years including direct fluorimetry, fluorescence polarization enzyme-linked immunosorbent assay (ELISA), and various biosensors and strip methods (Fernández-Cruz et al., 2010). The microbial contamination through fungal propagules is the major hurdle in providing hygienic and quality enriched food and feed products, and represents a prominent issue of food safety aspects, among the various industrialist and researchers (Garnier et al., 2017). The solutions for diminishing and/or preventing the fungal spoilage in food or diminishing the consumables are more challenging. However, the recent advances and development in food processing through employing different principles that include good manufacturing practices (GMPs) and hazard analysis of critical control points (HACCP) (Lockis et al., 2011; Cusato et al., 2013; Maldonado-Siman et al., 2014) have provided some relief to keep the final food products healthy and safe for consumption (Kumar et al., 2017). In the past few years, there has been a continuous rise in developing methods to prevent and control such contamination. Although the usages of some traditional methods employing physical tool and technologies are currently being used in common practices to prevent and control mold contaminations (Garnier et al., 2017). The other methods that have been frequently employed include good manufacturing and hygiene practices with the help of some rigorous control methods such as temperature control, reformed atmospheric packaging, efficient use of decontamination system, and air filters that have provided some positive hope (Garnier et al., 2017). Despite of having such technologies, still we do not have full control over fungal spoilage. Although, it has been suggested that the prevention of mold growth and mycotoxin secretion on plant and feed-stuff provide the best strategy for management of food spoilage problems (Blagojev et al., 2012) and to counteract the hazardous effects over human and animal health, the detoxification and decontamination of spoiled products is of prime importance (Blagojev et al., 2012). In recent years, considerable efforts have been focused on developing some preservation technologies (bio-protective cultures) for delimiting the food spoilage in food and feed products (Garnier et al., 2017). Furthermore, it was reported that prevention of food contaminants and mycotoxin production in food and feed products could be managed well efficiently through the application of biocontrol agents (Ribes et al., 2017). It was recently reported that lactic acid bacteria (LAB) has proven to be an efficient microbial antagonist and biocontrol agent among the other reported biological system, as it has been well demonstrated that LAB directly controls mold growth and have the potential for decontamination of mycotoxins through interaction (Blagojev et al., 2012). Apart from these, some natural agents (retrieved from plant, animals and microorganisms) or their derived products and other biocontrol microbes (*Bacillus, Streptomyces*, antagonistic yeast) have been successfully employed for preventing post-harvest decay caused by fungi (Ribes et al., 2017) as it was reported that *Geotrichum candidum* inhibits the growth and mycophenolic (MPA) production in *P. roqueforti*. Moreover, some novel strategies have been employed to reduce the drawbacks associated with antifungal agents including edible films and active packaging, incorporation into oil in water emulsions, nanoemulsions, edible films and active packaging, and their combination with other natural preservatives (Ribes et al., 2017).

The extensive perusal of literature revealed that the information on PR toxin is very scattered or limited. Therefore, the present review provides comprehensive and up to date informations including natural occurrences, diversity, biosynthesis, genetics, toxicological aspects, control strategies, and other management aspects of PR toxin with respect to food quality and safety. This review necessarily provides critical informations to the health-conscious consumers and other research professionals.

## Morphological Characteristics, Natural Occurrence, and Genetic Diversity

*Penicillium roqueforti* is a fast-growing fungus characterized by low and velutinous dark green colonies with moderate to heavy conidiation and either grayish turquoise or dull green coloration at colony margin. The reverse side of colony appears pale brown to green or deep blue-greenish coloration (almost black). The conidiophores originate from sub-surface hyphae with characteristic stripes, bearing large and dense brush-like spore-bearing structures (penicillii) at their terminal end, and characterized typically by having three staged branched (terverticillate) or occasionally more staged branched (quaterverticillate) spore-bearing system with having rough walls. Based on morphology, molecular data, and secondary metabolite production the *P. roqueforti* comprises three accepted species that includes *P. carneum*, frequently associated with meat, cheese, and bread, and produces patulin, penitrem A and mycophenolic acid (MPA). *P. paneum*, grow profusely with bread and silage, produces patulin and botryodiplodin; and *P. roqueforti*, could be isolated from various processed foods and silage and produces PR toxin, marcofortines, and fumigalclavine. The molecular characterization using PCR primer pairs and based on ITS region, it was reported that the 300 bp fragments obtained from specific primer could be used for identification of all the associated members belonging to *Penicillium* genera but also distinguishes *P. roqueforti* and *P. carneum* (Fernández-Bodega et al., 2009). The genotypic ribotyping of seventy-one different strains of *P. roqueforti* isolated and cultured from different starter cultures of blue cheeses were genotypically characterized using random molecular markers (RAPD) and were reported to bear high genetic similarity (Geisen et al., 2001). However, recently, it was found that significant morphological and genetical differences exist in between the large worldwide collections of *P. roqueforti* (Ropars et al., 2014; Gillot et al., 2015) which influenced their nominations for bearing Protected Geographical Indication (PGI) or identified by a Protected Designation of Origin (PDO) based on their utilities in cheese manufacturing types accordingly (Gillot et al., 2015) which reflects the presence of functional diversity exists among the different isolates of *P. roqueforti* (Gillot et al., 2017b). The isolates of *P. roqueforti* that have been recovered from baled grass silages have been reported to produce mycophenolic acids, and therefore consumption of such silages by livestock may become problematic for livestock producers. The predominant fungal species in grass silages is *P. roqueforti* that grows at low pH and microaerophilic conditions (Pahlow et al., 2003). Moreover, some characteristics such as resistance against organic acids and low pH provides favorable environment for flourished growth of *P. roqueforti* and therefore, found frequently as food and feed contaminants from various processed feedstuffs including bread, beer, rye bread, hard cheeses such as blue (Scott et al., 1977), blue-molded (Lafont et al., 1979), blue moldy tulum cheese (Erdogan and Sert, 2004), blue-veined (Moubasher et al., 1979) and olives, and produces toxic secondary metabolites like mycophenolic acid, PR toxin and its derived products. Interestingly, the majority (90%) of the *P. roqueforti* isolates consistently produces andrastin A and roquefortine C but differ significantly in their production for citreoisocoumarin, PR toxin, roquefortine A, and andrastin C (O’Brien et al., 2006). Due to their stability, the toxic metabolites such as roquefortine C and mycophenolic acid have been frequently found and recovered from grass silages. In contrast, the other including PR toxin and patulin are presumably unstable and therefore, their occurrences in blue cheese have not thought to pose a significant threat to the consumer’s safety.

## Chemistry, Stability, Degradation, and Biosynthesis of PR Toxin

**FIGURE 2:**
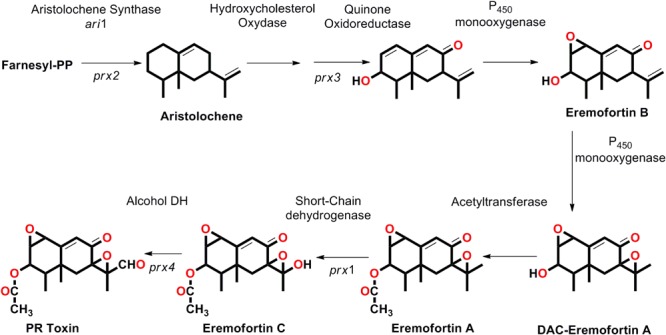
The step wise biosynthetic route, genes involved (*prx* gene clusters) that leads in the biosynthesis of PR toxin and its derived products (proposed by Hidalgo et al., 2014).

Chemically, the PR toxin is a bicyclic sesquiterpene having several functional groups including acetoxy (CH_3_COO^-^), aldehyde (-CHO), and α, β-unsaturated ketone (-C^α^= C^β^-CO) group with the presence of two stable epoxide rings, and belongs to eremophilane terpenoid class (Wei et al., 1975). The 17 C atom entire skeleton of PR toxin is derived from isopentyl diphosphate and an acetyl group. The PR toxin derives from the 15 carbon atoms sesquiterpene aristolochene and the reaction is catalyzed by an enzyme called aristolochene synthase (encoded by *ari1*: *prx2* gene) (Hohn and Plattner, 1989; Proctor and Hohn, 1993; Hidalgo et al., 2014). Further, the non-oxygenated aristolochene is converted to the trioxygenated intermediate EreB and catalyzed by three enzymes (i) a hydroxysterol oxidase-like enzyme (ii) quinone-oxidase that converts the bicyclic aristolochene nucleus into quinone type structure having C-8 oxo group and the C-3 hydroxyl group, and lastly (iii) P_450_ monooxygenase (epoxidase) that functions in epoxide formation by introducing double bond between C-1 and C-2. EreB, further oxidized by P_450_ monooxygenase for introducing a second bond between C-7 and C-11 prior to acetylation of EreA. Further EreA gets converted to EreC by oxidation of the side chain of the molecule at C-12 by an oxidase, a short chain oxidoreductase, and catalyzed by *prx1* (gene silencing studies confirmed the role of *prx1* in PR toxin biosynthesis in *P. roqueforti*). The last step of the biosynthetic pathway is characterized by an oxidation reaction at C-12 and catalyzed by a short chain alcohol dehydrogenase enzyme to form PR toxin (**Figure 2**; Hidalgo et al., 2014). The variation observed in the biosynthesis of PR toxin in different isolates of *P. roqueforti* is supposed to be due to the differential expression of genes that fine-tune and therefore, regulates the biosynthetic clusters encoding for PR toxin (Hidalgo et al., 2014).

Since the full genome sequence for *P. roqueforti* was not available earlier, the biosynthetic route for PR toxin was explored in *P. chrysogenum* (form PR toxin in traces) (Hidalgo et al., 2014) using gene cloning and RNA interference-mediated silencing, and to characterize the PR toxin cluster related gene in *P. roqueforti*. It was further predicted that PR toxin derives from sesquiterpene aristolochene, which is generated after the condensation and cyclization of three molecules of farnesyl diphosphate, a reaction catalyzed by aristolochene synthase (encoded by *ari1* gene) (**Figure 2**; Hidalgo et al., 2014). However, the RNA interference gene silencing studies confirmed the role of *prx* gene clusters (*prx1, prx2, prx3*, and *prx4)* adjacent to *ari1* gene as gene silencing of these *prx* gene clusters (*prx1* to *prx4*) resulted into a drastic reduction (65–75%) in PR toxin biosynthesis and increased formation of an antibiotic/immunosuppressant mycophenolic acid (Fernández-Bodega et al., 2009). This might be attributed to consume the accumulated C5 isopentenyl unit recovered from the decreased PR toxin synthesis (Hidalgo et al., 2014). The bioinformatics analysis revealed that in *P. chrysogenum* these four central genes had a high degree of identity (97–98%) to those of *P. roqueforti prx1* to *prx4*, and the *P. chrysogenum* homologue cluster contains 11 genes (Hidalgo et al., 2014). However, the data available from recent genome sequencing of *P. roqueforti* provided an in-depth insight into the comparative analysis of the genome organization in between *P. roqueforti* and *P. chrysogenum*. Based on the comparative data for PR toxin biosynthesis in both fungi, it was found and predicted that overall the *prx* gene clusters are similar in both fungi, although *prx5, prx6*, and *prx7* genes were reported to be located on different genomic segments away from other *prx1, prx2, prx3, prx4, prx8, prx9*, and *prx11* genes in *P. roqueforti* with no orthologs available for *prx10* in *P. roqueforti* (García-Estrada and Martín, 2016). The biosynthesis of PR toxin leads into formation of several other related and intermediate compounds such as eremophortins A, B, C, and D as revealed by the recent evidences on enzymes encoded by PR toxin gene clusters related to PR toxin named eremofortin A (EreA), eremofortin B (EreB), eremofortin C (EreC) and a tricyclic by product eremofortin D (EreD) an intermediate compound, formed in the biosynthetic route targeting for production of PR toxin (Moreau et al., 1980), and was also revealed from the enzymatic studies done on PR gene clusters (**Figure 2**; Hidalgo et al., 2014). In fact, new intermediates have been found and reported in the biosynthetic route available for PR toxin in *P. roqueforti* (Riclea and Dickschat, 2015). However, the biochemical conversion of eremophortin C to PR toxin could also be achieved through the activity of eremophortin C specific oxidases (EC). The molecular crosstalk that exists in between the pathways required for production of one secondary metabolite could lead into the biosynthesis of other compounds as it was described above the biosynthesis of mycophenolic acid by the gene silencing of PR toxin cluster genes. Similarly, the transcriptomic studies between two isolates of *P. chrysogenum* revealed that the mutant lacking the three biosynthetic genes responsible for the production of penicillin had shown the over expression of two biosynthetic genes *prx1* and *prx10* leading into the enhanced biosynthesis of PR toxin (Harris et al., 2009). These examples predict why certain strains produces specific metabolites while other not because, there exist a cross-regulatory pathways which allow the production of specific metabolites and negatively regulate/inhibit the other biosynthetic route (Bergmann et al., 2010; Hidalgo et al., 2014), as in the above example the biosynthesis of PR toxin is negatively regulated by an ORF locating inside the penicillin gene cluster. The unstability of PR toxin in culture medium due largely to the rapid conversion of PR toxin into related derivatives such as PR imine (more unstable product), PR amide (EreE), through reactions with amino group of the amino acids and catalyzed by PR amide synthase (**Figure 1**; Chang et al., 2004a,b). Therefore, we can conclude that PR acid is an oxidative product of PR toxin (Chang et al., 1998), and Ere represent the last intermediary compound in PR biosynthesis pathway, and that could also be confirmed from the observation as oxidation of EreC leads into formation of PR toxin.

## Dose-Response and Toxicogenetics of PR Toxin

### Microorganisms

The PR toxin is an aristolochene-derived bicyclic sesquiterpenoid compound with robust antimicrobial, genotoxic and cytotoxic activity due to its high chemical reactivity (Moulé et al., 1977; García-Estrada and Martín, 2016). The aldehyde group present in PR toxin structure is mainly responsible for its wide variety of biological effects, highlighting their toxicological properties (Cacan et al., 1977; Moulé et al., 1977). Hitherto, numerous negative effects of PR toxin on microorganisms have been reported by numerous researchers (Ueno et al., 1978; Polonelli et al., 1982). PR toxin was considered toxic for a ciliate protozoan *Colpidium campylum* at the minimal active dose 0.25 μg/ml (Dive et al., 1978). In contrast to PR toxin, EreA-D were not toxic to this ciliate protozoan. PR toxin has been proved to possess either direct or indirect genotoxic effects, causing genomic modifications and genetic mutations in microorganisms. Few studies showed its DNA-attacking ability in the rec assay on *Salmonella typhimurium* (Nagao et al., 1976; Ueno et al., 1978), and a mutagenic capacity in three strains of *Saccharomyces cerevisiae* and *Neurospora crassa* (Wei et al., 1979). Nevertheless, PR toxin also has significant inhibitory effects on RNA polymerase of *Escherichia coli* and on nuclear ribonuclease H activities of *Tetrahymena pyriformis* (Tashiro et al., 1979). Owing to this, Moulé et al. (1976) reported that during transcription process, the initiation as well as the elongation reaction of the RNA polymerase I and II of *E. coli* was affected by PR toxin exposure. PR toxin inactivates RNA polymerase and ribonuclease H probably through masking SH groups of the active center (Nakamura et al., 1977; Tashiro et al., 1979).

### Animals

Since, the discovery of PR toxin (C_17_H_20_O_6_) nearly 45 years ago, we have come across a plethora of different secondary metabolites related to PR toxin namely, PR acid (C_17_H_20_O_7_), PR imine (C_17_H_21_O_5_N) and PR amide (C _17_H_21_O_6_N) that have been isolated and studied in detail (Cacan et al., 1977; Moreau et al., 1977, 1980). Among these, only PR toxin is lethal and can cause a wide range of short-term as well as long-term ill-effects in organisms, ranging from immediate or delayed toxic response to potentially adverse long-term carcinogenic effects in animals such as rats, mice, and cats (Wei et al., 1975, 1976; Chen et al., 1982). It is worth mentioning that in spite of being well known for its acute toxicity, only a few toxicity data and its implicated mechanism are available in the literature (Hidalgo et al., 2014, 2017; Riclea and Dickschat, 2015; García-Estrada and Martín, 2016). Previous studies reveal that PR toxin is lethal to rodents after administered orally (p.o.), intraperitoneally (i.p.), or intravenously (i.v.) with LD_50_ values of 11.6 mg/kg (i.p.), 8.2 mg/kg (i.v.) and 115 mg/kg (p.o.) in rats, and 5.8 mg/kg (i.p.) in mice (Wei et al., 1973, 1976; Chen et al., 1982; Scott, 1984). Polonelli et al. (1978) studied the toxicity of PR toxin in rats using i.p. administration and found that it developed abdominal writhing, decreased respiratory rate, motor incoordination, flaccid paralysis, especially in the hind legs and ataxia with impaired or altered immune response, which may lead to death. The LD_50_ value was 14.5 mg/kg body weight. Other symptoms due to exposure of PR toxin include ascites fluid and oedema in the scrotum and lungs, and an increased number of white blood cells (WBCs) in rodents and cats, and resulted in an increase of pleural volume and pericardial fluid (Chen et al., 1982). These effects were largely due to an increase in capillary or microvascular permeability resulting in direct damages to blood vessels and organs such as lungs, heart, liver, and kidneys (Chen et al., 1982). In addition, it was found to be cytotoxic and affect liver cell viability at a very low concentration in rat liver (Aujard et al., 1979). Collectively, these data suggest that PR toxin was hepato-and nephrotoxic to mice. PR toxin is believed to be responsible for outbreaks of mycotoxicosis in livestock that had ingested *P. roqueforti*-contaminated silage (Wei et al., 1973). Further, this toxin exposure has also been associated with intestinal irritation, ruminal stasis, reduced feed intake, liver toxicity, bovine abortions, reduced fertility and retained placenta with immunotoxic properties in cattle (Still et al., 1972; Wei et al., 1984; Berge, 2011). In a previous experiment, Veselý and Vesel (1981) fed corn silage infected with *P. roqueforti* to dairy cows which resulted in poor appetite, cessation of rumen activity, decreased fecal output, gut mucosal inflammation as well as abortion. In particular, high producing livestock can especially be subject to subacute symptoms due to PR toxin mycotoxicosis (Gallo et al., 2015). However, evidence of adverse negative health effects due to PR toxin ingestion in ruminants is still scarce, uninformative, controversial and inconclusive (Storm et al., 2008; Whitlow and Hagler, 2010). In a recent study, it was shown that unlike other *P. roqueforti* toxins, PR toxin did not interfere with *in vitro* rumen fermentation parameters, supporting the hypothesis that rumen microbial ecosystem has an effective capacity to degrade or inactivate the PR toxin into less toxic products. Thus, PR toxin seems to be less hazardous to ruminants than other tested mycotoxins (Gallo et al., 2015). Finally, even though relationship between PR toxin and livestock disorders is controversial and not fully explored (Pedrosa and Griessler, 2010; Whitlow and Hagler, 2010), any effect due to ingestion of *P. roqueforti*-contaminated cereals/forages on ruminal digestive and metabolic responses need to be investigated thoroughly.

PR toxin has inhibitory effects on vital cellular processes as well, such as protein and RNA synthesis (Moulé et al., 1976, 1978). Specifically, it interferes with the activities of chromatin-bound DNA-dependent polymerases α, β, and γ (Lee et al., 1984; Wei et al., 1973, 1976, 1985) as well as normal process of mitochondrial respiration (oxidative phosphorylation) by inhibiting HCO_3_^-^ATPase activity in rat brain, liver, kidney, and heart (Wei et al., 1984; Hsieh et al., 1986). On another hand, PR toxin derivative (e.g., PR imine) has diminished, inhibitory effects on protein and nucleic acid synthesis (Moulé et al., 1977), and were shown to exhibit less toxicity in mice after acute exposure (Arnold et al., 1978). The LD_50_ for mice is in the range of 100 to 200 mg/kg by i.p. administration. Similarly, other degradation metabolites namely, EreC failed to cause death or abnormalities in mice dosed 10 mg/kg body weight (Moreau and Moule, 1978). These effects are largely due to the structural difference between the EreC and PR toxin specifically at the C-12 position, the most important position for at least the *in vivo* toxic effect. Concerning genotoxicity studies, PR toxin induces formation of DNA-protein cross-links in chromatin structure of both cultured cells as well as isolated rat hepatic nuclei and found to be carcinogenic for rats (Polonelli et al., 1982). Rats fed PR toxin developed adenocarcinoma, squamous epithelioma, and a uterine sarcoma, which were shown histologically.

### Humans

Despite the fact that PR toxin is able to impact many hepatic, intestinal, neurological, and immune functions in animals, there appear to be virtually no reports on potential health hazard associated with PR toxic in humans (Engel and von Milczewski, 1977; Leistner and Eckardt, 1979). However, the increased interest in the mycotoxin research during the last few years has drawn the scientific attention on evaluating their toxicity on human cell lines. Hitherto, PR toxin was reported to be cytotoxic in porcine and human cell lines (Aujard et al., 1979; Lompe and von Milczewski, 1979). In this respect, Rasmussen et al. (2011) determined the *in vitro* cytotoxicity of PR toxin extracted from fungal agar and maize silage. Using resazurin assay, it was found that the PR toxin like other major mycotoxins significantly affected the human intestinal epithelial cell (Caco-2) viability and the IC_50_ value was in the range of 1–13 μg/mL. Based on the above findings PR toxin could be considered as most acute cytotoxic metabolite derived from *P. roqueforti*. Recently, Hymery et al. (2017) investigated the effects of PR toxin on two different proliferating state cell cultures, namely, the human intestinal epithelial cells (Caco-2) and monocytic immune cells (THP-1) through an *in vitro* study to understand the precise mechanisms involved in its toxicity. In this respect, the cytotoxicity studies showed a dose-dependent effect of PR toxin and the calculated mean cytotoxic concentration (IC_50_) concentrations for Caco-2 and THP-1 cells were >12.5 and 0.83 μM, respectively. Moreover, tumor necrosis factor (TNF)-α, interleukin (IL)-1β and IL-8 genes involved in the inflammatory reaction were studied. Gene expression studies showed that tumor necrosis factor (TNF)-α expression was significantly increased after exposure in THP-1 cells as compared to Caco-2. Further, the cell culture assay showed a 10-fold reduction in PR toxin concentration, indicating that PR toxin was degraded by THP-1 cells (most sensitive cells). Collectively, these data suggest that PR toxin appears to be one of the most cytotoxic on Caco-2 and/or THP-1 cells and induces both necrosis and an inflammatory response in THP-1 cells. Therefore, it is worth mentioning that, from a cognitive point of view, it is obvious that the need for PR toxicological studies must be increased and more emphasis must be placed on human health rather than animal feed sanitation.

The commercially exploited strains of *P. roqueforti* used as starter cultures and other those recovered from blue cheeses have the ability to produce PR toxin (Orth, 1976; Wei and Liu, 1978; Engel and Prokopek, 1979; Medina et al., 1984; Chang et al., 1991; Boysen et al., 1996; Geisen et al., 2001). Noteworthy, the microaerophilic conditions and presence of nitrogen compounds such as amino acids, casein, amines and ammonium salts provides the unstable condition for the production of PR toxin in blue cheeses, which presumably degraded to various derivatives, namely PR acid, PR imine and PR amide, that are considered as less toxic molecules (Vallone et al., 2014; Hidalgo et al., 2014; Riclea and Dickschat, 2015). These compounds, unlike the PR toxin and its Ere derivatives, can be found in blue cheeses (Moreau et al., 1980; Chang et al., 1993). However, there is some evidence that in *vivo* (mice), PR imine is reversibly converted into more toxic compound, i.e., PR toxin (Moulé et al., 1977). This may pose a potential health and safety risk for humans. However, human health effects from PR toxin in association with cheese have not been reported (**Figure 1**; Pitt and Hocking, 1997). Similarly, the other PR toxin related metabolites found in Auvergne blue cheese, named EreA-D obtained from a strain of *P. roqueforti* were also not acutely toxic to mice at respective doses of 15, 15, and 50 mg/kg (i.p.) and did not inhibit protein and RNA synthesis (Moreau et al., 1976; Arnoux et al., 1977; Moulé et al., 1977; Cacan et al., 1978).

Finally, over the years, all the above-mentioned studies highlighted the toxic effects of PR toxin on microorganisms, animals, and human cell lines but little is known about their toxicity and mode of action on plant cells. Further, despite their toxicological interest, little progress has been made in the last decade to elucidate the biosynthesis and molecular genetics of PR toxin (Hidalgo et al., 2014; Riclea and Dickschat, 2015; García-Estrada and Martín, 2016).

## Analytical Methods for Determination of PR Toxin

Accurate sampling method before analysis is one of the most critical and crucial factor for achieving a satisfactory verified analysis of PR toxin contamination in silage or grain samples (Rundberget et al., 2004; O’Brien et al., 2006; Rasmussen et al., 2010, 2011). The first step for detection of PR toxin involves proper sampling of silages or grains, which poses the main source of variation or random errors (∼90%) associated with quantification could be co-related with how the original representative sample was collected. This is often a difficult task as *P. roqueforti* can produce percentage of mycotoxins in small areas called hotspots or pockets, making the very low concentrations of PR toxin extremely variable in their distribution within the bulk lot of silages or grains. In addition, the presence of *P. roqueforti* does not, in fact, necessarily mean that the related PR toxin will also be occur, as its formation is largely affected by interactions with certain other environmental factors (Möller et al., 1997; Pedrosa and Griessler, 2010; Berge, 2011). In the same manner, the absence of *P. roqueforti* will neither assure freedom from PR toxin as the fungi may have already perished while leaving the toxin intact in the representative sample. To overcome these drawbacks, it is important to develop accurate sampling methodology to ensure that the analyzed sample must be a representative of the whole lot or consignment and give a meaningful result during PR toxin analysis. Following sample collection, the whole primary sample must be ground, minced or homogenized into extremely smaller particles in order to get a more uniform concentration of toxin for the analytical test. Depending up on the particle-based size, a subsample is taken for PR toxin extraction. Owing to this, the various analytical testing methods can be used toward the proper detection of toxin following a traditional subsample pretreatment process which generally involves the extraction of toxin via an adequate extraction mixtures such as water/acetone, chloroform or acetonitrile (Chang et al., 1993; Erdogan et al., 2003; Pascale and Visconti, 2008) through liquid–liquid extraction (LLE), supercritical fluid extraction (SFE) and solid phase extraction (SPE) or solid-liquid extraction (SLE) approach, followed by a clean-up process to eliminate undesired extracted interference from the toxin, which may include sample concentration, and finally the purification through separation as well as the detection using analytical equipments (Pascale and Visconti, 2008). Recently, several developed chemical extractive methods such as QuEChERS or dispersive liquid-liquid microextraction (DLLME) have been used for an effective determination of mycotoxins, particularly for multi-mycotoxin methods; however, conventional techniques mentioned above such as solid phase extraction are still probably the extractive procedures which are most widely used (Jelen, 2002; Pereira et al., 2014; Leggieri et al., 2017).

In the recent years, there have been significant developments in a broad range of modern analytical as well as detection techniques that can be applicable, useful and practical for the determination of PR toxin. Several reviews have highlighted recent developments in practical approaches and new analytical methods, which offer flexible and broad-based methods of analysis and detection of compounds in silages, cereals and related foodstuffs (Krska et al., 2005; Shephard, 2008; Cigić and Prosen, 2009; Reiter et al., 2009; Turner et al., 2009; Fernández-Cruz et al., 2010; Pereira et al., 2014). However, there is no official technique for analysis and/or detection, due to the variety of other closely related structural secondary metabolites to PR toxin such as PR acid, PR imine and PR amide and its precursor viz. EreA-E (Hidalgo et al., 2014; Riclea and Dickschat, 2015). Also, rapid, sensitive and reliable analytical methods are unavailable due in part by a deficiency in surveillance data for less known mycotoxins such as PR toxin.

Over the years, various widely applicable separation methods such as thin layer chromatography (TLC), liquid chromatograph (LC) commonly linked with mass spectrometry (LC-MS), ultra-performance liquid chromatography with tandem mass spectrometry (UPLC-MS/MS), high performance liquid chromatography (HPLC), gas chromatography (GC), gas chromatography-mass spectrometry (GC-MS), capillary electrophoresis, supercritical fluid chromatography and other novel techniques were used for the separation and quantitation of PR toxin in forages, silages, grains and related foodstuffs. Unfortunately, the traditional chromatographic techniques for separation are usually time-consuming, labor or capital intensive, and therefore, expensive, hence a variety of antibody-based immunochemical methods, have been produced and commercialized for rapid analysis/detection (Meulenberg, 2012; Matabaro et al., 2017). These methods include enzyme-linked immunosorbent assay (ELISA), radioimmunoassay (RIA), chemiluminescence immunoassays (CL-IA), fluorescent immunoassays (FIA), lateral flow immunoassay (LF-IA), flow injection immunoassay (FIIA), and various biosensors and strip methods (Fernández-Cruz et al., 2010). These techniques are robust, simple, specific, highly sensitive, easy-to-handle, a high degree of flexibility, easy to analyze and cost-effective, and in some cases portable methods for routine analysis, which have been extensively used as detection tools for detecting infectious agents, pathogens or in screening analysis of PR toxin in wide range of samples. Recently, however, research has implicated the ongoing need for the utilization of very specific biosensors in real-time, reproducible to a high level, field-portable devices and instruments for the detection and identification of infectious agents, pathogenic microorganisms, toxins, and other contaminants in foods and many other items (Dubey et al., 2017; Meena et al., 2017). Despite, most of the immunochemical methods are lab-based, generally applicable for only single mycotoxin or small group of its structurally relevant compounds. Unlikely, the conventional chromatographic techniques can separate a plethora of analytes including some with a drastically distinct chemical nature or structure. Indeed now a days, chromatographic techniques, such as LC, GC, and HPLC coupled to MS detector, after chemical derivatization, are the most frequently used analytical techniques for multi-toxin analysis and confirmation purposes of PR toxin (Jelen, 2002; Sørensen et al., 2007; Mioso et al., 2015; Leggieri et al., 2017). However, the screening of samples is commonly performed by using TLC or immunoassay-based methods like ELISA, just allowing qualitative or semi-quantitative results (Siemens and Zawistowski, 1993; Kononenko et al., 2015). Although, previously TLC and HPLC chromatographic techniques were the most commonly used analytical methods for the simultaneous detection of PR toxin (Wei et al., 1979; Siemens and Zawistowski, 1993; Möller et al., 1997; Chang et al., 1998; Erdogan et al., 2003; Nielsen et al., 2006; O’Brien et al., 2006; Fernández-Bodega et al., 2009). However, recent advancement and development in analytical instrumentation have highlighted the potential of LC-MS/MS, particularly for multi-toxin determination purpose is gaining much popularity (De Santis et al., 2017; Kim et al., 2017; Laganà, 2017). Recently, Rodríguez et al. (2017) reviewed the specific features for the analysis of natural toxins by LC and LC-MS, while Gillot et al. (2017a) provided the specific features for analysis of PR toxin and related compounds via LC-MS/Q-TOF. Importantly, Brock and Dickschat (2013) successfully applied a newly developed trace analytical technique that combines all the advantages of closed-loop stripping apparatus (CLSA)/GC-MS technique with those of NMR spectroscopy for the detection of hitherto unrecognized derivatives, side products, and intermediates of the PR toxin biosynthetic route. However, to the best of our knowledge, there is currently no single detection technique available that can stands out above the rest to meet the requirements, for the determination PR toxin in forages, silages, cereals, and related foodstuff products.

## Control and Management of PR Toxin Contamination

Several control strategies have been developed to eliminate or avoid the harmful effects of PR toxin since its discovery in the 1970s. In this respect, a good agronomic practice has been shown to have a most profound and contrasting effect on the management of PR toxin contamination of silages as well as cereal crops in the fields. The key ecological determinants during the pre-and post-harvest practices are the climatic conditions, particularly, water availability (moisture content) and temperature. Other extrinsic major factors, includes poor pre-harvest practices, time and handling produce during harvesting, improper storage and transportation, marketing and processing, composition and bioavailability of micronutrients in the substrate, relative humidity, exposure time, sanitation, aeration, plant vigor, insect infestation, damage to seeds, invertebrate vectors and other attack from other pests with surveillance, management and awareness creation, can also actively facilitates mold growth and increase the risk of PR toxin contamination problem (Zain, 2011; Gallo et al., 2015; Wambacq et al., 2016, 2018; Wambacq, 2017). Unlike extrinsic factors, the intrinsic factors comprising of strain specificity and prevalence, strain abundance, variation, spore loads, microbial interaction and unstability of toxigenic properties are often more difficult to monitor (Chang et al., 1991; Boysen et al., 2000; O’Brien et al., 2006; Dell’Orto et al., 2015). Accurate and consistent information is therefore needed in this regard toward the impact of an association between these key extrinsic and intrinsic factors, and it is essential to recognize which are marginal and which critical for mold proliferation and PR toxin production. Hitherto, there have been limited attempts to consolidate the available information on these crucial factors in relation to PR toxin and contaminated commodities such as silages and cereals. However, prevention of mold and PR toxin contamination during pre/post harvest or storage has not been found satisfactory.

On another hand, contaminated food and feeds can be eliminated, inactivated or detoxified by using physical, chemical and biological control strategies depending upon the conditions. At present, there are several methods based on these strategies to inhibit mold growth, eliminate or reduce the PR toxin levels, degrade or detoxify the grains and grass silages. However, the best possible way to abstain the presence of PR toxin in food and feedstuff is to prohibit the fungal contamination, since there is an array of limitations associated with the degradation or detoxifications. The utilization of biological detoxification agents, such as microorganisms and their enzymatic products, or natural sources like essential oils and herbal extracts to avoid growth of mycotoxigenic fungi on contaminated food and feed can be a choice of such technology. From a cognitive point of view, more attention should be paid to understand the degradative pathway of the PR toxin, the structure of biotransformed products and the enzymes responsible for the detoxification. Indeed, in these cases, PR oxidase, PR-amide synthetase and eremofortin C oxidase from *P. roqueforti* catalyze the conversion of PR toxin to PR acid, PR acid into PR amide, and EreC to PR toxin respectively can serve as a potential candidate (Chang et al., 2004a,b; García-Estrada and Martín, 2016). Unfortunately, these approaches used in food commodities have their limitations or disadvantages, which limit their large-scale application (Milićević, 2010). Specifically, none of the above methods meets the criteria of Food and Agriculture Organization (Beaver, 1991). Therefore, it is expected that progress in the control of PR toxin contamination will depend on the introduction of new technologies and advances for specific, efficient and environmentally sound detoxification. The following control strategies can be effectively applied for the inactivation or decontamination/of PR toxin.

### Physical Methods

A plethora of traditional methods or traditional hurdle technologies are implemented alone or in combination to prevent and control PR toxin contaminations. One of the common approaches frequently employed as physical sorting of the mold contaminated grains based on their properties (mainly physical aspects) or through the use of fluorescence methods manually. While a number of other methods include cleaning and washing, milling, winnowing, adsorption, heat treatment, extrusion, solar, UV, γ-rays, X-rays or microwave irradiation, anaerobiosis and extraction of toxins were effective in achieving significant PR toxins removal (Suttajit, 1989; Scott, 1998; Sengun et al., 2008). In general, PR toxin is moderately stable during most of these physical processes and persists into finished foods and silages. Interestingly, heat and ultraviolet light application are reported that are effective methods to reduce PR toxin content in silages and food grains. However, since fungal contamination and toxin production may occur at any stage from pre-harvest in the field to crop storage and food processing, the complete elimination is not achievable.

### Chemical Methods

Chemical methods have been widely used as the most effective means for the inactivation or detoxification of PR toxin from contaminated commodities. A variety of chemical approaches such as acid/base treatment, ozonation, deamination, oxidizing/reducing agents, ammoniation, chlorinated agents, formaldehyde, and so on have been used for degrading or inactivating the PR toxin in contaminated commodities (Jard et al., 2011; Benkerroum, 2016). The most widely used chemical preservatives with potential antifungal properties in industries as food additives are inorganic compounds, e.g., sulfite, nitrite, and ammonium sulfate; weak organic acids, such as acetic, propionic, lactic, sorbic and benzoic acid; antioxidants such as selenium, beta-carotenes, vitamins A, C, and E (Lind et al., 2005; Ashiq, 2015; Yan et al., 2017). These chemical food additives are mainly restricted to specific categories of foods and feedstuffs, or they are onerous due to huge cost, problems and practical difficulties involved in the R&D and product development. Nevertheless, the appropriate use of these chemical preservatives during the pre-harvesting and packaging process could help in preventing the fungal infection and consequently mycotoxin contamination in a wide range of food and feed products. However, use of some chemical preservatives or fungistatic chemicals is being discouraged now a days due to economic reasons and growing awareness of environmental crisis and food safety issues. Further, during the course of evolution, *P. roqueforti* has acquired the ability to resist chemical and some preservatives treatments such as organic acids. For example, *P. roqueforti* isolates have been found to acquire resistance against benzoate (Cabo et al., 2002). Owing to this phenomenon, there is a great future concern that the resistance will increase due to more frequent use of the vast majority of antibiotics and preservative chemicals. Further, due to many of other deficiencies such as mutagenicity and deleterious change in the treated raw product, the methods have not become popular for its full-fledged large-scale commercial application. Therefore, the use of anthropogenic chemical treatment methods in feed and food products has been already banned in the European Union. Similarly, mycotoxin binders or adsorbent products used as a feed supplement to mitigate mycotoxins in livestock diets are not approved by FDA for the prevention or treatment of mycotoxicoses. Despite being several mycotoxin adsorbent materials are generally recognized as safe (GRAS). Finally, in the recent years, there has been a significant advancement in nanotechnology particularly in the field of nanoencapsulation as well as the expansion of its application in various food preservative sectors including mycotoxin detoxification through adsorption processes or photocatalytic degradation (Prakash et al., 2018).

### Biological Methods

*Penicillium roqueforti* infestation is often found in the variety of food commodities, where they can cause extensive damage and leads to colossal economic losses. This fungus leads to food spoilage such as off-odors, off-flavors development, discoloration, decay, and disintegration of the food structure. The prevalence of their toxic metabolites – PR toxin – constitutes a higher safety risk for human and animal health. Although, prevention of mold growth and its toxin production considered being the best strategy to impede their harmful effects, inactivation/detoxification of contaminated commodities is also of prime importance. Interestingly, some strains in the recent time posses unique ability to resist against certain chemical treatments and preservatives. Nevertheless, potentially adverse environmental impacts and health effects of certain fungicides and preservatives led to a search for more natural methods. Use of plant varieties with increased *P. roqueforti*-infection resistance, avoiding stress and damage to plant can be another alternative. However, as the population demands higher quality, secure, non-toxic, non-preservative, less processed food with prolonged shelf life of product, the biopreservatives or natural occurring essential oils and plant extracts, has received ample attention recently. Valerio et al. (2017) through their preliminary studies also emphasized the importance of antimold competing microorganisms and plant metabolites as natural fungicides and their inhibitory effects against *P. roqueforti* having potential use in intelligent food packaging. However, such inhibition may not significantly alter the possible toxic effects of the toxin. Among the different potential decontaminating or detoxify competing microorganisms, the group of the lactic acid bacteria, *Bacillus, Streptomyces*, and yeast has been considered as the most promising natural biological antagonists for food safety (Dalié et al., 2010; Blagojev et al., 2012; Laref and Guessas, 2013). Alternatively, antifungal agents or secondary metabolites produced by these organisms have a great potential to be used as natural biological control agents in food preservation to minimize the growth and mycotoxin production by molds. On the other hand, smart packaging via eco-friendly biodegradable biofilm integrated with naturally bioactive compounds obtained from these organisms as a bio-preservative represents a new frontier.

### Lactic Acid Bacteria (LAB)

The antifungal activity of LAB has been attributed due to a wide variety of active antagonistic metabolites namely, organic acids, carbon dioxide, ethanol, hydrogen peroxide, fatty acids, acetoin, diacetyl, cyclic dipeptides, low molecular mass polypeptides, bacteriocins, or bacteriocin-like inhibitory substances (Höltzel et al., 2000). The action of antifungal properties within LAB on some mycotoxigenic molds have been reported by several authors (Dalié et al., 2010; Crowley et al., 2013; Russo et al., 2016), but the number of published studies on the antifungal activity of LAB toward *P. roqueforti* is still very scared. Despite, it has been well known for decades that ensiling or fermenting forages with LAB are relatively simple yet effective way of preserving forage for future use by livestock (Pedrosa and Griessler, 2010).

Valerio et al. (2009) tested many strains of LAB against *P. roqueforti*, a general contaminant of bakery products. The results have shown that *P. roqueforti* inhibited by almost all the tested strains at a percentage higher than 65.52% and the most effective strain was found to be *Lactobacillus plantarum* C21–41. Further, the antifungal activity of these bacterial strains was found to be comparable with that obtained from common chemical preservative calcium propanoate or calcium propionate (C_6_H_10_CaO_4_). Similarly, Yan et al. (2017) also assessed the *in vitro* antagonistic potential of *L. plantarum* against *P. roqueforti* as an indicator and the effect of its antifungal activities in an increase in the shelf life of Chinese steamed bread. The *in vitro* screening of antifungal activity was verified by double-layer plate point inoculation, hyphal radial growth rate assay, conidial germination assay, and agar diffusion. All the ten tested LAB strains significantly inhibited the growth of *P. roqueforti* and were shown to have different inhibitory activities. Out them, only *L. plantarum* CCFM259 (CCFM259) exhibited good inhibition of mold mycelial growth and conidial germination, with inhibition rates of 45.3 and 18.0%, respectively. On another hand, according to their results, the water/salt-soluble extract of *L. plantarum* also had antifungal activity toward *P. roqueforti* CCFM259. In contrast, prior studies that used *P. roqueforti* DPPMAF1 as an indicator of antifungal activity reported that 40.5% conidial germination and the hyphal radial growth rate of spoilage mold were inhibited by the water/salt-soluble extract of sourdough fermented wheat germ (SFWG) fermented with *L. plantarum* LB1 and *L. rossiae* LB5 (Rizzello et al., 2011). As previously shown, the inhibitory effects in the LAB extract was attributed due to the organic acids such as lactic acid, acetic acid, malic acid, phenyllactic acid and citric acid fermented by *L. plantarum* (Laitila et al., 2002; Lavermicocca et al., 2003). These results are in agreement with the work of Lavermicocca et al. (2000) who reported that the culture filtrate of *L. plantarum* grown on wheat flour hydrolysate showed a strong inhibitory effect toward the growth of *P. roqueforti* IBT18687. Therefore, it is unlikely that this phenomenon is due to antifungal activity, which would result in an increased preservation of Chinese steamed bread. In a recent study, 88 *L. plantarum* strains were assessed for their antifungal property against *P. roqueforti* CECT 20508 and *P. chrysogenum* CECT 2669 (Russo et al., 2016). The overlayed method was used for a preliminary discrimination of the strains while those isolates that displayed broad antifungal spectrum activity were further screened based on their antifungal properties in cell-free supernatant (CFS). The results revealed that in contrast to other LAB strains, *L. plantarum* UFG 121 had the strongest antifungal potential and found out to be a most potent strain. The foregoing results suggested that lactic acid was produced at high concentration during the growth phase, indicating that this metabolic aptitude, associated with the low pH, contributed to explain the highlighted antifungal phenotype of the isolate. In an another study, Zhang et al. (2015) characterized a strain of *L. plantarum* TK9 isolated from the Chinese naturally fermented congee for its probiotic and food preservation potential. The strain exhibited a broad range of antifungal spectrum against *P. roqueforti* and its other closely related food spoilage species. In addition, to further evaluate its bio-preservative potential, *L. plantarum* TK9 was inoculated into citrus, apples, and yogurt prior to the addition of molds. The results indicated that *L. plantarum* TK9 prolonged the shelf life of all the tested food and represented as an effective candidate for food-related bio-preservative. In a similar study, Coda et al. (2011) investigated the antifungal activity of wheat sourdough bread LAB using *P. roqueforti* DPPMAF1 as the indicator to extend their shelf life. However, unlike the above results, the antifungal activity of the selected *L. plantarum*1A7 (S1A7) was marked to be lower toward *P. roqueforti* DPPMAF1. Similarly, the antifungal LAB has also shown an activity spectrum which excluded *P. roqueforti* (Magnusson et al., 2003; Cheong et al., 2014). Despite these facts, it is worth mentioning that compared to the antifungal efficacy of selected metabolites products of the LAB, DL-3-phenyllactic acid (PLA) at 7.5 mg/ml exhibited the excellent antifungal activity against *P. roqueforti* (mainly *Penicillium* spp.) that spoil cheddar cheese (Manu, 2012). Collectively, these data suggest that PLA has significant potential to inactivate molds in shredded cheddar cheese and can offer new perspectives for use as a fungicidal preservative, especially in dairy products. However, still a few limited numbers of reports shown that a good selection of LAB strains could prevent the growth of *P. roqueforti* and therefore reduce or eliminate the health risks associated due to their mycotoxins exposure in already contaminated products (Dalié et al., 2010).

### Bacillus

Notably, *Bacillus subtilis* is well-known for producing antifungal compounds such as Alboleutin, Bacitracin, Botrycidin, Clorotetain, Fengycin, Iturins, and Rhizocticin which prevent or inhibit the growth of a large number of fungi as well as yeasts. Whereas most of these antifungal metabolites have been screened out against fungal mycelial growth and only scanty of reports are available about their effect on spore survival and germination of *P. roqueforti*. Chitarra et al. (2003) isolated, characterized and identified an antifungal compound from *B. subtilis* YM 10-20 which checked the germination of *P. roqueforti* conidiospores, often a contaminant of bakery and silage products. Using different techniques, it is shown that this iturin A like compound efficiently permeabilizes and inhibits the germination of conidiospores. In a previous study, Babad et al. (1952) also reported a polypeptide produced by *B. subtilis* with antifungal activity against *P. roqueforti* under *in vitro* condition. In a recent study, it was shown that the antifungal properties of organic extracts obtained from *B. amyloliquefaciens* VJ-1 were effective against *P. roqueforti* and *P. chrysogenum* under *in vitro* investigation (Kadaikunnan et al., 2015). In contrast, Lavermicocca et al. (2003) reported that 3-phenyllactic acid produced by *Bacillus* strains exhibited strong inhibitory effects against a wide range of molds, including *P. roqueforti*. The bacterium *B. velezensis* was found to exhibit significant antagonistic activity toward the development of *P. roqueforti* sensu lato (s.l.), the most prevalent fungal contamination in silages (Wambacq, 2017). Both culture supernatant and suspension of the *B. velezensis* was found to reduce the spore germination, their survival and mycelia growth of the mold. Interestingly, these results seem very promising toward the management and control of contaminated silages. However, future research is required via *in vivo* microsilo trial to investigate the antagonism potential of *B. velezensis* as a silage additive to counter *P. roqueforti* s.l. and facilitating its applications in silage management.

### Streptomyces

The prolonged preservation to extend the shelf life of food and feed products could also be obtained via *Streptomyces* species. The extensive perusal of literature suggests that few species of *Streptomyces* were reported to inhibit the growth of resistant strains of *P. roqueforti*. For example, Frändberg et al. (2000) evaluated the antifungal potential of a compound extracted from *Streptomyces halstedii* K139 as a potent inhibitor of the *P. roqueforti* as well as toward the range of other potentially harmful molds. However, the inhibitory effect of compounds isolated from *Streptomyces* have certain limitations as their efficacy is well dependent on the target fungal species used against, and in some cases were found to be insufficient for controlling the growth of mold species.

### Yeasts

Hitherto, several researchers observed that live yeast culture was shown to reduce the detrimental effects of *P. roqueforti* in contaminated feed and food. For example, Lillbro (2005) examined the antagonistic potential of several yeast strains against *P. roqueforti* growth in a miniature grain silo with a moist wheat grain. The inhibiting effect of yeast strains was significantly enhanced through the production of killer toxin against *P. roqueforti*, with the addition of different carbon sources to the miniature silo. *Pichia farinosa, Candida silvicultrix, P. guillermondii, P. burtonii, C. fennica, C. pelliculosa, C. lusitaniae*, and *C. silvicola* were found out to be a potential candidate in air tight storage of cereal grain. In a similar study, Petersson and Schnürer (1995) investigated the antagonistic potential of *Pichia anomala, P. guilliermondii*, and *Saccharomyces cerevisiae* to inhibit the growth of the mold *P. roqueforti* in non-sterile high-moisture wheat grains. It was found that *P. anomala* had the strongest and most potent antagonistic activity in wheat, completely inhibiting the growth of *P. roqueforti*. Further, the inhibition of *P. roqueforti* was greatly affected by the extrinsic factors and found to be least pronounced at the optimum temperature (21°C) and water activity (0.95). Moreover, the inhibition activity varied among isolates, *P. guilliermondii* slightly reduced the growth while *S. cerevisiae* inhibited the mold growth weakly at the highest inoculum level. Juszczyk et al. (2005) supported the above observation that most of the yeasts effectively slowed down the growth rate of *P. roqueforti* strains, as their application provided good results and were characterized by the reduction in conidia number/Petridish, with a delayed time of sporulation. Surprisingly, it was found that among all yeast species used against *P. roqueforti*. However, the best results in terms of inhibitory response were given by strains of *C. lipolytica* exhibiting a strongest inhibitory effect toward growth as well as sporulation of *P. roqueforti*. On another hand, there are several other reports that yeast *Yarrowia lipolytica* inhibits the growth and sporulation of *P. roqueforti* (Devoyod, 1990; van den Tempel and Jakobsen, 2000; Cantor et al., 2004; Romano et al., 2006). The biocontrol activities of yeast species against *P. roqueforti* have several limitations as their inhibitory action was found to be reduced at salt concentration (5%) and interaction between the two was highly strain dependent and delimited by water activity (aw).

Many previous studies have shown that the yeast *P. anomala* can inhibit mold growth and sporulation on agar plates as well as in high-moisture grain stored under airtight systems (Petersson and Schnürer, 1995; Boysen et al., 2000). In this respect, Druvefors et al. (2005) unravelled the mechanism underlying the inhibition of grain spoilage mold *P. roqueforti* using *P. anomala* J121 as biocontrol yeast which is due to the production of sugar metabolites via glycolysis under airtight storage condition. Despite, the competition for oxygen and high levels of carbon dioxide might be the other mechanisms that contribute to the antifungal activity. The discovery that sugar amendments enhance the antifungal activity of *P. anomala* suggests novel ways of formulating the antagonistic yeasts.

In a recent study, the comparative evaluation between the antifungal activities and technological properties of three doughs or sourdoughs that were fermented by *Wickerhamomyces an*omalus LCF1695 (formerly known as *Hansenula anomala* or *P. anomala*) and *L. plantarum* 1A7 against *P. roqueforti* DPPMAF1 were reported using agar diffusion, growth rate inhibition, and conidial germination assays (Coda et al., 2011). The results revealed that dough fermented by *W. anomalus* LCF1695 (D1695) showed the most effective control of the indicator organism when compared with *L. plantarum* 1A7. Based on foregoing results, *P. anomala* is, therefore, considered as an attractive alternative for biocontrol to enhance the storage stability of high-moisture grains. Other features that make *P. anomala* as a robust microorganism suitable for effective biocontrol is its ability to grow and survive at wide range of temperatures (3°C – 37°C), pH (2–12.4), carbon and nitrogen sources under oxygen-limited or depleted conditions (Fredlund et al., 2002). Further, the potential of mycocinogenic yeast to inhibit spoilage mold *P. roqueforti* in yogurt is another novel approach with a potential to minimize yogurt spoilage and extend its shelf-life. Liu and Tsao (2010) determined the effectiveness of mycocinogenic yeast *Williopsis saturnus* var. *saturnusas* B9043 as a biocontrol agent against spoilage mold *P. roqueforti* in plain yogurt samples. *W. saturnus* var. *saturnus* B9043 inhibited the growth of tested mold in a dose-dependent manner. The filtrate of *W. saturnus* var. *saturnus* B9043 showed good inhibitory results against *P. roqueforti* and reflects the inhibitory role of mycocin. These results provide sufficient facts and information that the antifungal activity of this yeast is highly dependent on initial spore concentration and crucial for effective biocontrol aspects.

### Essential Oils and Herbal Extracts

Due to mutagenic, teratogenic and carcinogenic side effects of chemical preservatives used in foods treatments, the demand for healthy foods with fresh ingredients, or at least less processed foods have risen. This caused to develop research methodologies for substitution of chemical preservatives by adding natural ingredients or compounds derived from plants to prevent fungal growth and toxin production (Mossini and Kemmelmeier, 2008; Sajadi, 2015). Several researchers had reported the inhibitory effect of various plant extracts and oils on mycotoxin production and fungal growth (Zargar, 2014). Due to this reason, the application of natural ingredients against *P. roqueforti* was evaluated by several researchers and gaining attention in recent years. In a previous study, Azzouz and Bullerman (1982) reported a moderate antifungal activity of crude drug isolated from oregano against *P. roqueforti*, when applied in a concentration of 2% (the optimum level that is most often used in the food processing industries). On another hand, an octanoic acid derived from coconut and palm kernel oil can prevent the growth and PR toxin production in *P. roqueforti* (Jelen et al., 2002). In this context, Araujo et al. (2009) reported the *in vitro* antifungal activity of alcoholic extracts derived from different spices on the growth and development of *P. roqueforti*, a common contaminant in homemade bread. The results showed the significant inhibitory effect of alcoholic extracts from dehydrated plants on the mycelial growth and sporulation of *P. roqueforti*, except for garlic. Similarly, Pereira et al. (2006) evaluated the antifungal effect of ten powdered spice plants to observe their inhibitory effects on mycelial development and sporulation of *P. roqueforti*. The results revealed that unlike others, clove had significant potential for complete inhibition of the mycelial growth of the mold under study which is also evidenced and supported through several other earlier studies (Hitokoto et al., 1980; Karapinar and Aktug, 1987; Farag et al., 1989; Bara, 1992). In the recent year, the antimicrobial properties of twenty-five essential oils and plant extracts toward *P. roqueforti* NRRL 849 contamination in pet food products have been investigated (Bianchini et al., 2014). The activity was tested *in vitro* and the mold was best inhibited by cinnamon essential oil (0.01%) and spearmint essential oil (0.5%). Likewise, other spices including, cinnamon, garlic, thyme, mint, anis, oregano and basil also have been tested and shown to have promising antifungal activity. When tested in the extruded product (either mixed into the product or as part of its coating), both most promising essential oils, cinnamon (0.05% and 0.1%) and spearmint essential oil (0.5%) proved ineffective against *P. roqueforti*. Collectively, these data suggests that spice essential oils can act as inhibitors of *P. roqueforti* in pet food products, when present in an optimal concentration. Similarly, Matan et al. (2006) reported the anti-mold potential of cinnamon oils toward *P. roqueforti* indicating that cinnamon is a natural antimicrobial agent. Indeed, Sørensen et al. (2010) reported that the antimicrobial activity of oat seed extract is due to the effect of chitinase I, an antifungal enzyme, employed especially for controlling the growth of *P. roqueforti* mold. Moreover, the chitinase I extracted from oat seed extracts (compared to wheat, barley, and rye) were found to be 10 times more abundant and effective in controlling the growth of mold. Further, Šimović et al. (2014) tested oregano and clove essential oil components carvacrol and eugenol for their antifungal effect against foodborne pathogenic *P. roqueforti* PTFKK29 under *in vitro* and *in situ* conditions for improving the safety of fresh-cut watermelon. Both the selected components showed excellent inhibitory activity against *P. roqueforti* PTFKK29. Therefore, the study provides scientific evidence to prefer natural alternatives such as eugenol and carvacrol over other chemical preservatives used for fresh cut and ready to eat fruits to improve the quality of fruit products and other food safety issues. In an another study, Akgul and Kivanç (1988), reported the efficacy and activity of some selected Turkish spices against the foodborne *P. roqueforti* and reported the strong inhibitory effects of oregano ground essential oil when compared to sorbic acid. On the other hand, Valerio et al. (2017) tested the antifungal activity of twelve bacterial, fungal and plant-derived metabolites against common fungal food contaminant the *P. roqueforti* and concluded that the plant metabolites α-costic acid and ungeremine showed the highest and potent inhibitory activity. Thus, these plant metabolites appear to be a suitable candidate for their inclusion in a biodegradable biofilm aimed to obtain a new material for an ‘intelligent’ food packaging. However, such type of attempts has to be tested toward the efficacy of other medicinal plant aqueous extracts against the growth of *P. roqueforti* and to restrict their subsequent PR toxin production.

## Economic Impact and Legislation of Pr Toxin

The disruption and deterioration of food consumables through microbial resources, particularly mycotoxins is one of the most important concerns among all the challenging issues raised for the food safety issue. The worldwide mycotoxin contamination of foods and feedstuffs is a recurring problem for safety purposes across the globe. The economic impact of the mycotoxins on human health, animal productivity and trade could be determined and evaluated based on multiple criteria. The surveillance studies have shown extensive PR toxin contamination in silage, grains, or other food and feedstuff products in both developing and developed countries (Miller, 2008; Driehuis et al., 2008; Storm et al., 2010; Driehuis, 2013; Kononenko et al., 2015). The presence of PR toxin in these products, particularly in the silages is of high safety risk concern potentially for human and animal health due to their properties to induce deleterious toxicity effects at relatively low doses (Sumarah et al., 2005; Pedrosa and Griessler, 2010; Storm et al., 2010; Rasmussen et al., 2011; Gallo et al., 2015). Therefore, its contamination and implications are important to consider in high producing livestock, both for their economical management, trade as well as for maintaining the health and productivity of the cattle. The significant considerations include animal life, health hazards and veterinary care costs, reduced livestock production, regulatory cost, expenditure done on research studies to assess the severity and detoxification strategies for PR toxin. In addition, PR toxin produced from fungi grown on cereals was significantly higher in contrast to Ere and PR toxin by fungi grown on legumes (Chang et al., 1991). Due to these reasons, the PR toxin-induced economic damages becomes a significant problem in all the food sectors and more specifically it affects the economy of food sectors dealing with consumption and production of grains products. Interestingly, because of their potential occurrence in staple foods, PR toxin is of considerable significance from the public health viewpoint. Contrary, PR toxin and their health effects on humans is in its infancy and much more are waiting to be discovered. Extensive searches for non-toxic strains for use as cheese starter cultures have so far been largely unsuccessful (Pitt, 1997). However, to our knowledge, no international regulation exists, in the EU or other countries for maximum PR toxin level in feed materials. The legislation for the regulations of mycotoxin contamination in food products has been already approved in more than 100 countries across the globe. In contrast, the legislation for the microbiological status of animal feed in most countries including EU counties has neither documented nor have any written legislative limits available till date. Therefore, continued international efforts are needed to set guidelines or practical measures for effective control of PR toxin, and to implement it adequately.

## Concluding Remarks

The increasing health awareness among the world growing population and the public interest for the demand of quality enriched food products have influenced the various sectors of food biotechnology, employing microbial entities for feed and food processing, to produce nutritious and health-promoting food products. The rapid increase in microbiological food safety outbreaks are the results of mass production, a globalized food trade, and intricated food supply chains. The increased global demand for food has made this market more competitive to improve the safety of their products, avoid microbial contamination and other losses relevant to the commercial production that affects their economy. Since, the consumption of PR toxin enriched food have acute toxic effects over the health of both human and animals, we need to search strategies through which one could prevent the product contamination. Biological control and other natural methods have provided, a more effective and indigenous strategy for regulating the toxic strains of *P. roqueforti*. Moreover, the comprehensive knowledge of biosynthetic route for production of PR toxin, gene regulation and the genome sequencing data could reveal the necessary and critical informations for regulating the genetic machinery of this fungus in a favorable manner, to eliminate the steps leading to production of PR toxin or modify the overall pathway for synthesis of bioactive compounds. Today there is an intense need for developing strains with altered biochemical pathways for their safe and efficient exploitations at commercial scale in various sectors of food industries. Additionally, we must have certain legislation to regulate the safe usages of this fungus for food security and other biosafety challenges issues.

## Author Contributions

MD conceived and drafted part of the manuscript. MD, MA, and MK wrote the manuscript. SK and MM helped in editing, formatting as well as critical review of manuscript. SS and RU supervised the current study. All authors read and approved it for publication.

## Conflict of Interest Statement

The authors declare that the research was conducted in the absence of any commercial or financial relationships that could be construed as a potential conflict of interest.
